# Supplementary material for: Effectiveness of kaolin-impregnated hemostatic gauze use in preperitoneal pelvic packing for patients with pelvic fractures and hemodynamic instability: A propensity score matching analysis
Source: PLoS One. 2020 Jul 24;15(7):e0236645. doi: 10.1371/journal.pone.0236645 (PMC7380609; doi:10.1371/journal.pone.0236645)
Supplement: Attachment — Submitted filename: Rebuttal letter.docx [file pone.0236645.s001.docx]

Dear. Editor in chief and review.

Thank you for your comments. I trust that this manuscript will be more fruitful by your comments. We really appreciate it. We revised this manuscript point to point. Thank you.

**The authors are required to respond to the reviewer's comments and to add a brief about their standard of care of patients with pelvic ring injury associated with haemodnamic instability. This will explain the use of HG in the study group and the role of angio-embolization.**

--> Thank you for your comment. It's good opinion. I appreciate it. According to our guideline for pelvic bone fracture with hemodynamically unstable based on the Rocky Mountain Regional Trauma Center at Denver Health, we perform PPP and/or EF and if bleeding is ongoing, perform pelvic angiography, which is 2ndary following PPP. I explained it little bit more in method section. In addition, there is no criteria for the use of hemostatic gauze and it was determined by surgeons. I add it in method section.

**Although the authors discussed the small sample size as a limitation of the study, they did not mention the cause of including only 53 out of 75 patients with PPP.**

--> Yes. Actually, small sample size is a limitation of this study. Among 75 patients, we excluded 22 patients. Because PPP didn't undergo for these 22 patients. We explained that in patient selection and data collection section before. I highlighted it sky blue color.

**1. Please ensure that your manuscript meets PLOS ONE's style requirements, including those for file naming. The PLOS ONE style templates can be found at http://www.plosone.org/attachments/PLOSOne_formatting_sample_main_body.pdf and http://www.plosone.org/attachments/PLOSOne_formatting_sample_title_authors_affiliations.pdf**

-> We did it according to your comment. Thanks.

**2. Please provide additional details regarding participant consent. In the ethics statement in the Methods and online submission information, please ensure that you have specified (1) whether consent was suitably informed and (2) what type you obtained (for instance, written or verbal). If your study included minors under age 18, state whether you obtained consent from parents or guardians. If the need for consent was waived by the ethics committee, please include this information.**

-> We did it accordiing to your comment. Thanks.

3. Thank you for stating the following financial disclosure:

"The funders had no role in study design, data collection and analysis, decision to publish, or preparation of the manuscript."

At this time, please address the following queries:

a.Please clarify the sources of funding (financial or material support) for your study. List the grants or organizations that supported your study, including funding received from your institution.

b.State what role the funders took in the study. If the funders had no role in your study, please state: “The funders had no role in study design, data collection and analysis, decision to publish, or preparation of the manuscript.”

c.If any authors received a salary from any of your funders, please state which authors and which funders.

**d.If you did not receive any funding for this study, please state: “The authors received no specific funding for this work.”**

-> I put this comment "The authors received no specific funding for this work."

5. Review Comments to the Author

Please use the space provided to explain your answers to the questions above. You may also include additional comments for the author, including concerns about dual publication, research ethics, or publication ethics. (Please upload your review as an attachment if it exceeds 20,000 characters)

Reviewer #1: The authors studied in a retrospective study a comparison between HG and CG in PPP for hemodynamically unstable pelvic fractures. They utilized propensity matching technique.

**The co-primary outcomes were the rate of occurrence of postoperative wound infections and hemorrhage induced mortality (does not exactly reflect the title)**

--> We changed the study title to ‘Safety and effectiveness of Kaolin-impregnated hemostatic gauze use in preperitoneal pelvic packing for patients with pelvic fractures and hemodynamic instability: propensity score matching analysis’.

**They demonstrated no differences in wound infections between the two groups, although the number of patients was too small to really conclude this (by the way-high rate of infections…). Beside the need for a larger N, since the focus of the study id wound infection, a detail of the organisms involved would be useful.**

-> Thanks for comments. Therefore, I add this sentence. "Identified microorganisms in the HG group were Staphylococcus epidermidis, Staphylococcus epidermidis and Enterobacter aerogenes for one patient, and Enterococcus faecalis. Staphylococcus epidermidis, E.coli, and Methicillin resistent Staphylococcus capitis were identified in the CG."

**Cardiac arrest in the ER was included- were these patients successfully resuscitated and taken to the OR?**

--> Yes. We included those who were successfully resuscitated in the ER.

**12 patients received angiography and angioembolization? What was the distribution between the groups?**

--> According to the protocol, secondary pelvic angiography following PPP was performed in cases of ongoing bleeding even if PPP was performed. Embolization procedures were performed in cases of contrast media extravasations during pelvic angiography. Pelvic angiography was performed for 5 patients in the HG group and 4 patients in the CG (table 4). Pelvic angioembolization was performed for 2 patients in the CG, and 1 patient in the HG group (out of table).

**Is it your institution practice to use either HG or CG at surgeon discretion? Since this is a retrospective study one cannot determine if the choice of treatment was based on severity of patient condition therefore may introduce a treatment bias.**

--> Yes. When performing PPP, there were no criteria for the use of hemostatic gauze and it was determined by surgeon. But, it wasn't based on severity. The usage of hemostatic gauze was used randomly. We used propensity scoring matching, because there are some differences of characteristics in both groups which is definitely limitation of retrospective study.

**Figure 2, I would recommend to delete.**

--> Thank you for the comment. I will do it.

**In general, absent a protocol/guideline on when to use PPP vs angio etc.. it is difficult to conduct a retrospective study and in addition to a very small cohort.**

--> Thanks for your comment. Actually we have protocol. I mentioned it in material and method section.

The management protocol for pelvic fractures with hemodynamically unstable was initiated in our trauma center in May 2014 according to management protocols created by the Rocky Mountain Regional Trauma Center at Denver Health (12).

According to the protocol, secondary pelvic angiography following PPP was performed in cases of ongoing bleeding even if PPP was performed. Embolization procedures were performed in cases of contrast media extravasations during pelvic angiography. In cases requiring external fixator (EF) application, pelvic binders were not re-applied; rather, these were re-applied just after PPP, but only in cases without EF use. Pelvic binders were removed after patients became hemodynamically stable.

Thank you again. We hope that you can keep safe in the situation.

Sincerely,

Ji Young Jang, M.D.

Department of Surgery, Trauma Center, National Health Insurance Service Ilsan Hospital

100 Ilsan-ro, Ilsan-donggu, Goyang-si, Gyenggi-do, Republic of Korea

Tel: +82-31-900-3624

E-mail: [drjangjiyoung@gmail.com](mailto:drjangjiyoung@gmail.com), [jyjang@hanmail.net](mailto:jyjang@hanmail.net)
