# Supplementary material for: Effectiveness of kaolin-impregnated hemostatic gauze use in preperitoneal pelvic packing for patients with pelvic fractures and hemodynamic instability: A propensity score matching analysis
Source: PLoS One. 2020 Jul 24;15(7):e0236645. doi: 10.1371/journal.pone.0236645 (PMC7380609; doi:10.1371/journal.pone.0236645)
Supplement: Attachment — Submitted filename: Author response.docx [file pone.0236645.s002.docx]

Dear. Editor in chief and review.

Thank you for your comments. I trust that this manuscript will be more fruitful by your comments. We really appreciate it. We revised this manuscript point to point. Thank you.

Reviewer #1: Although the authors modified the title to reflect the subject of their results, the primary outcome remains wound infections and as such this manuscript does not discuss or present adequate data on infections. For example a table on microorganisms, antibiotics, localized infection vs sepsis etc...

Again the size of this study is a major limitation.

- We agree with the reviewer’s opinion and exclude wound infection from the primary outcome. Accordingly, the contents related to wound infection were removed from the manuscript and ‘safety’ was also excluded from the title of the study.

In addition, I fully agree with the reviewer’s comments on the small study population. However, in this study, propensity score matching was performed to compensate for the problems of the small sized retrospective study, and the normality test of the two groups was checked to confirm the normal distribution of the two groups to confirm the statistical adequacy. So, we think our paper is unique, as there are no previous studies on the use of hemostatic gauze in patients with PPP. The results of this study are also meaningful in that they present on hypothesis for future randomized controlled studies. Through this, I think we can expect that a better hemostatic effect in patients undergoing PPP.

Thank you very much for your important review.

Reviewer #2: I would like to thank the authors for their article and the valuable information. I have the following comments and questions.

I think that the major concerns from the readers side about your work would be:

1. Lines 90 - 101: The primary aim of your study was to evaluate the safety and effectiveness of kaolin-impregnated hemostatic gauze in PPP for patients with hemodynamic instability due to severe pelvic fractures. Between lines 90 - 101, you were describing your protocol for the management of patients with hemodynamic instability and pelvic fractures. You described the use of pelvic binders and external fixation according to the preference of the orthopaedic surgeons in the trauma team. When you analysed the results of kaolin-impregnated hemostatic gauze versus the control group PPP, we can't find data about how many patients had external fixation in each arm and how many patients had only pelvic binders. I think this is a very important piece of information to see if there any significant difference in the rates of external fixation vs pelvic binders in each treatment group. Furthermore, we can't find any data about the types or technique of external fixator, if it was C-clamp, supra-acetabular anterior external fixation, etc.

🡪 Although orthopedic surgeons in our hospital decided whether or not to perform external fixation, it was performed in only 18.9% of unstable patients due to the lack of trauma-dedicated orthopedic surgeon in Korea. Thus, our protocol includes the application of the pelvic binder in the emergency room in all pelvic fracture patients with shock. External fixation application rates in both groups were added to the manuscript (Table 1,2,4). All external fixation was supra-acetabular external fixation and C-Clamp was not used in our trauma center. We added this contents to the material and method section.

2. Lines 150 - 161: Regarding the patients' characteristics in your study, there were 17 patients LC-II (32%) and one patient LC-I. Hemodynamic instability with pelvic fractures are known to be significantly prevalent in pelvic fractures APC-III, LC-III and VS according to the data published by Young and Burgess in 1990 and according to our personal experience. However in your patient group, you have more patients with LC-II injuries compared to VS (30%) and APC-III (7.5%). Can you please explain this?

🡪 Authors agree with the reviewer’s opinion. There are several reasons for the high proportion of LCII in our cohort and relatively low proportion of APCII and APCIII.

First, because the trauma system in our area has not fully organized, 68% (36/53) of patients have been transferred from other hospitals to our trauma center, and the mean time from accident to ER arrival was 156 minutes, which is quite long. We think that Hemodynamically unstable patients with more severe pelvic fracture such as APC III or VS died prior to hospital arrival.

Second, in our cohort, the pedestrian TA was 41.5% and the average age of patients was 60, which is relatively high compared to other cohorts. For these reasons, pelvic fracture caused by low energy injury often cause shock. These also seems to be the reason for the high proportion of LCII pelvic fracture patients in our cohort.

Other comments:

3. Line 71: Your study is a retrospective analysis or prospectively collected data and according to the patients and methods (Because the data were analyzed anonymously, informed consent was exempted.), however in line 71, you state in your inclusion criteria (3. agree to the collection and use of their medical information.). If the informed consent was exempted.

🡪 Third inclusion criterion was wrong, so we deleted it.

4. Lines 195 -198: I think details of identified micro-organisms in both treatment groups can be removed. Most important was the rates of deep infection. Review Comments to the Author

Please use the space provided to explain your answers to the questions above. You may also include additional comments for the author, including concerns about dual publication, research ethics, or publication ethics. (Please upload your review as an attachment if it exceeds 20,000 characters)

Reviewer #1: Although the authors modified the title to reflect the subject of their results, the primary outcome remains wound infections and as such this manuscript does not discuss or present adequate data on infections. For example a table on microorganisms, antibiotics, localized infection vs sepsis etc...
Again the size of this study is a major limitation.

Reviewer #2: I would like to thank the authors for their article and the valuable information. I have the following comments and questions.

I think that the major concerns from the readers side about your work would be:

1. Lines 90 - 101: The primary aim of your study was to evaluate the safety and effectiveness of kaolin-impregnated hemostatic gauze in PPP for patients with hemodynamic instability due to severe pelvic fractures. Between lines 90 - 101, you were describing your protocol for the management of patients with hemodynamic instability and pelvic fractures. You described the use of pelvic binders and external fixation according to the preference of the orthopaedic surgeons in the trauma team. When you analysed the results of kaolin-impregnated hemostatic gauze versus the control group PPP, we can't find data about how many patients had external fixation in each arm and how many patients had only pelvic binders. I think this is a very important piece of information to see if there any significant difference in the rates of external fixation vs pelvic binders in each treatment group. Furthermore, we can't find any data about the types or technique of external fixator, if it was C-clamp, supra-acetabular anterior external fixation, etc.

2. Lines 150 - 161: Regarding the patients' characteristics in your study, there were 17 patients LC-II (32%) and one patient LC-I. Hemodynamic instability with pelvic fractures are known to be significantly prevalent in pelvic fractures APC-III, LC-III and VS according to the data published by Young and Burgess in 1990 and according to our personal experience. However in your patient group, you have more patients with LC-II injuries compared to VS (30%) and APC-III (7.5%). Can you please explain this?


Other comments:

3. Line 71: Your study is a retrospective analysis or prospectively collected data and according to the patients and methods (Because the data were analyzed anonymously, informed consent was exempted.), however in line 71, you state in your inclusion criteria (3. agree to the collection and use of their medical information.). If the informed consent was exempted.

4. Lines 195 -198: I think details of identified micro-organisms in both treatment groups can be removed. Most important was the rates of deep infection.

🡪 Yes, I agree with the reviewer’s comments and have deleted this.

Thank you for your thoughtful review.
